# Physical activity, interleukin-6 change, and gait speed

**DOI:** 10.18632/aging.204797

**Published:** 2023-06-03

**Authors:** Francesco Panza, Carlo Custodero, Vincenzo Solfrizzi

**Affiliations:** 1 Dipartimento Interdisciplinare di Medicina, University of Bari Aldo Moro, Italy; 2 Unit of Research Methodology and Data Sciences for Population Health, National Institute of Gastroenterology "Saverio de Bellis", Research Hospital, Bari, Italy

**Keywords:** physical activity, interleukin-6, gait speed, older adults, randomized clinical trial

Aging can be associated with a decline in physical function that eventually leads to a loss of autonomy in the activities of daily living (ADL). In particular, mobility is the most studied and most relevant physical ability affecting quality of life with strong prognostic value for disability and life expectancy [1]. In fact, walking is a component of ADL, and it is important for the main determinants of quality of life in older age such as maintaining independence in ADL, enjoying an adequate level of social interaction, and retaining good emotional vitality [2]. In older adults, gait speed has been described as the ‘sixth vital sign’ because it is a core indicator of health and function in aging and disease [2]. In fact, gait speed is an objective measure of physical functioning, with slower performances associated with mobility disability and other adverse health-related outcomes in older age [2]. Gait speed lower than 0.8 m/sec may be a reliable cut-off to identify subjects at increased risk for disability, hospitalization, institutionalization, and increased mortality [3], while improvement of usual gait speed may ensure a longer survival in older adults [4]. Gait speed measured over long distances (400 m) is a good indicator of cardio-respiratory fitness, and it may be a better early indicator of the overall physical health compared to gait speed over short distances [2].

In older age, one of the best and safe approach for improving gait speed is a multicomponent exercise intervention (e.g., including aerobic, muscle strengthening and balance training). However, not all the subjects undergoing physical activity intervention respond in the same way in term of improvement of physical function and cardio-respiratory fitness. Therefore, it is mandatory to determine the underlying biological mechanisms by which physical activity may affect 400 m gait speed, to better understand key pathways involved in determining mobility disability. Among biological factors involved in regulating the impact of physical activity intervention on gait speed, myokines, signalling molecules produced and secreted by skeletal muscle, may play a pivotal role in mediating the positive muscular and systemic effects of exercise training, by their autocrine, paracrine, and/or endocrine action . In older age, among myokines, interleukin (IL)-6 is a well-accepted marker of systemic inflammation and chronic low-grade inflammation has been recognized as one of the potential underlying causes of age-related diseases, associated with physical inactivity and recognized as an independent risk factor for incident disability, impaired mobility, and mortality [5].

Evidence on the relationship between circulating IL-6 levels and gait speed suggested that higher IL-6 levels may be associated with poorer performance in older adults and an increased risk of major mobility disability [5], defined as the self-reported inability to walk 400 m. However, as for most of biological processes, it is conceivable the possible presence of a not perfect linear relationship also between inflammation marked by IL-6 levels and physical performance. Different changes in elevation or reduction of plasma IL-6 levels might evoke different responses to physical activity intervention in term of gait speed. A post-hoc analysis from Lifestyle Interventions and Independence for Elders (LIFE) Study, a multicenter single-blind randomized clinical trial (RCT) on 1,300 sedentary older adults (mean age:78.85 ± 5.23 years,65.85% women) at risk for mobility disability investigated the effects that dynamic changes of IL-6 may have on gait speed during physical activity intervention [6].

This RCT demonstrated that a 12-month structured, moderate-intensity multicomponent physical activity intervention compared to a healthy educational intervention was associated with a significant benefit on 400 m gait speed in mobility-limited older adults in whom plasma IL-6 levels underwent to a yearly change between −1 and +2 pg/ml [6]. The effect size was greater than 0.2, then it should not be considered negligible. In a sensivity analysis, lower functioning older participants (Short Physical Performance Battery < 8) with a yearly reduction of IL-6 levels from −1 to −2 pg/ml experienced a significant worsening of gait speed (−0.082 m/sec), but no significant difference was estimated between physical activity and healthy educational interventions. Probably, frailer subjects with loss of homeostatic capacities leading to excessive reduction of IL-6 levels, likely related to anti-inflammatory drugs use or immunosuppression, might experience greater and faster worsening of functional performances, but further evidence are needed to confirm this hypothesis. Finally, in the LIFE Study, physical activity intervention did not significantly modify plasma IL-6 levels compared to healthy educational intervention after 12 months follow-up [6].

For the first time, this RCT reported that a IL-6 change confined between −1 and +2 pg/ml over one year follow-up, in association with multicomponent exercise training program, might produce a significantly greater gait speed over long distances (between 0.042 and 0.048 m/sec), both compared to subjects in the physical activity intervention group with other changes in IL-6 values, and those in the control group with the same yearly IL-6 change [6]. This gait speed difference might be also clinically significant in term of prevention of mobility disability. Indeed, changes in gait speed of 0.04–0.06 m/sec have been associated with clinically meaningful modifications in functional limitation [7].

Present findings may suggest the presence of an hormetic window for inflammatory state variations, marked by changes in IL-6 values, which could warrant better responses to physical activity intervention in terms of gait speed. Therefore, inflammation which is physiologically a protective response of human body to cope with endogenous and environmental stressors including exercise, may reflect a detrimental process when becomes dysregulated in amplitude and duration, i.e., during aging. Inflammation could represent a type of hormetic response also to exercise, in which repeated, transient and mild-intensity stressors may generate beneficial effects. In older adults with preserved adaptation capacity, exercise-induced IL-6 response may be an important mechanism silencing inflammatory pathways activated during age-related diseases, allowing better benefits on functional performance and potentially slowing down aging process. However, this hypothesis should be verified in *ad-hoc* studies with repeated measures of inflammatory markers and longer follow-up. Very recently, an animal study compared four groups of mice: sedentary old male mice, animals that received recombinant IL-6 (rIL-6) in an exercise-mimicking pulsatile manner, mice that were trained with a moderate-intensity, low-volume endurance exercise regimen, and mice that were exposed to a combination of these two interventions (rIL-6 and endurance exercise) for 12 weeks [8]. In this study, mice exposed to enhanced levels of IL-6 during endurance exercise showed superior improvements in endurance performance, fatigue resistance *in situ*, motor coordination, and gait following training in comparison with the other three mice groups [8]. These results in old mice suggested that a low-volume, moderate-intensity endurance training could be combined with the application of rIL-6 to achieve synergy in improving the functional capacity of skeletal muscle, particularly gait speed.

In mobility-limited older adults, a moderate intensity, structured physical activity intervention may produce a small but clinically meaningful benefit on 400 m gait speed compared with healthy educational intervention, when associated with variations of IL-6 between −1 and +2 pg/ml. However, further studies are needed to confirm these findings and test association betweenIL-6 changes during exercise and effects on other physical performances, metabolic parameters, cardiovascular and respiratory functions.
